# Assessment of skin fibrosis in a murine model of systemic sclerosis with multifunctional optical coherence tomography

**DOI:** 10.1117/1.JBO.30.3.036007

**Published:** 2025-03-27

**Authors:** Harshdeep Singh Chawla, Yanping Chen, Minghua Wu, Pavel Nikitin, Jessica Gutierrez, Chandra Mohan, Manmohan Singh, Salavat R. Aglyamov, Shervin Assassi, Kirill V. Larin

**Affiliations:** a University of Houston, Biomedical Engineering, Houston, Texas, United States; b University of Texas Health Science Center at Houston (UTHealth Houston), Division of Rheumatology, Department of Medicine, Houston, Texas, United States; c University of Houston, Mechanical and Aerospace Engineering, Houston, Texas, United States; d Baylor College of Medicine, Integrative Physiology, Houston, Texas, United States

**Keywords:** skin fibrosis, biomechanics, elastography, optical coherence tomography, angiography

## Abstract

**Significance:**

Systemic sclerosis (SSc) is a chronic idiopathic disease that causes immune dysregulation, vasculopathy, and organ fibrosis that affects more than 3 million people in the US alone. The modified Rodnan skin score (mRSS) is the current gold standard for diagnosing and staging skin fibrosis in SSc. However, mRSS is subjective, requires extensive training, and has high observer variability.

**Aim:**

We aim to provide a quantitative method for the assessment of fibrosis.

**Approach:**

We utilized optical coherence tomography (OCT), its extensions, optical coherence elastography (OCE), and OCT angiography (OCTA) to evaluate SSc-like fibrosis and therapy response in a mouse model.

**Results:**

We showed stiffness differences between fibrotic and normal mouse skin by week 4 (p=0.02) during the longitudinal study. In the treatment response study, OCE recorded higher elastic wave velocity in untreated fibrotic skin (p=0.04). Treated fibrotic skin stiffness was between normal and fibrotic levels. OCTA indicated significantly dilated microvasculature in fibrotic skin versus control (p≪0.01), with more dilation in the treatment group (p≪0.01) than in normal skin.

**Conclusions:**

Our results indicate that OCT and its extensions effectively analyze dermal fibrosis. OCE revealed increased stiffness in fibrotic skin, OCTA showed vessel dilation, and OCT noted morphological changes in fibrosis tissue.

## 1 Introduction

Systemic sclerosis (SSc), also known as scleroderma, is a chronic autoimmune condition primarily marked by skin and internal organ fibrosis.^1^ Despite its rarity (400 cases per million or 150,000 cases in USA), SSc poses significant clinical challenges because over half of SSc patients succumb to the disease within a decade post-diagnosis.^2^ Beyond its substantial mortality rate, SSc drastically deteriorates functionality and overall quality of life.^3^^,^^4^ One of the key barriers to the lack of Food and Drug Administration (FDA)-approved treatments for SSc is the inability of current methods to provide reliable quantitative measurements of skin involvement in SSc.

At present, the severity of skin involvement in SSc is gauged with the modified Rodnan skin score (mRSS)^5^ because dermal thickening and fibrosis are recognized as a defining characteristic of SSc.^6^^,^^7^ This method involves pinching and rolling skin at 17 specified body sites and assigning scores based on perceived skin thickness. Despite being the benchmark, mRSS has significant pitfalls—it is subjective, necessitates intensive training, and has high inter-observer variability.^8^ As mRSS is based on palpation, measurements can be skewed by the intrinsic physical characteristics of the skin, including elasticity. This has, in part, contributed to the absence of an FDA-endorsed therapy specifically for SSc-associated dermal fibrosis. There is an obvious and palpable need for an objective, repeatable, and precise tool to assess skin fibrosis, which would revolutionize drug discovery and patient care by paving the way for effective treatment modalities.

Various imaging methods have been proposed for skin fibrosis assessment, and high-frequency ultrasound techniques are currently the most popular,^9^ but other imaging modalities, such as magnetic resonance imaging, have also been utilized.^10^ However, each method comes with limitations, such as depth penetration, resolution, accuracy, or cost. On the other hand, optical coherence tomography (OCT) offers a promising balance between penetration depth (∼1 to 2 mm in the skin) and resolution (∼10  μm),^11^ positioning it as an ideal tool for detailed skin analyses.^12^ For example, OCT can see the epidermis-dermis junction (EDJ), enabling quantitative evaluation of skin layer thickness in SSc patients. Coupled with its ability to gauge tissue properties such as optical attenuation and scattering,^13^ OCT can become a useful tool for quantitative skin evaluation. Beyond structural imaging, OCT also has powerful functional extensions, most notably non-invasive mechanical imaging with OCT-based elastography, i.e., optical coherence elastography (OCE),^14^^,^^15^ and label-free vasculature imaging with OCT angiography (OCTA).^16^

Biomechanical skin alterations due to SSc could also serve as a diagnostic and monitoring biomarker because SSc progression is also marked by changes in tissue stiffness in addition to morphology. To that end, several modalities have been proposed to assess the biomechanical properties of SSc-afflicted skin. A device called the Vesmeter can assess the elasticity and viscosity of skin based on an indentation-based principle, and has been utilized for characterizing SSc involvement in the skin.^17^ However, this device requires specific pressure and point-wise measurements, which introduces approximately 20% inter-observer variability. Another method that has been rapidly gaining popularity is ultrasound elastography, which utilizes ultrasound imaging to detect displacements in tissue, either through manual compression^18^ or by inducing transversely propagating elastic waves.^19^ However, these techniques lack the resolution to simultaneously detect the structural and elastic characteristics of SSc-affected skin. In addition, clinical ultrasound elastography is performed by hand on the patient skin, which can significantly affect the reliability and repeatability of the measurements.^20^ To overcome these limitations, we have developed an OCE technique for assessing skin stiffness based on imaging the tissue response to mechanical wave propagation. This approach has been previously demonstrated to be highly repeatable^21^^,^^22^ without the need for a coupling medium because it relies on noncontact stimulation^23^ of the tissue.

In addition to skin thickness and stiffness changes, the microvasculature is also significantly altered due to the tissue remodeling caused by SSc.^24^ Techniques such as nailfold capillaroscopy have been used to detect vasculature changes in SSc patients.^25^ However, nailfold capillaroscopy can only provide limited information as it cannot acquire three-dimensional information and is also limited to the nailbed. Given that SSc can be highly heterogeneous, imaging the nailfold only may be insufficient. The functional angiographic extension of OCT, termed OCTA, is a powerful, label-free, non-invasive tool for imaging blood vessels in tissues, including the skin.^16^ Recent research has shown that OCTA could detect nailfold capillary abnormalities in SSc patients with higher sensitivity than traditional capillaroscopy.^26^^,^^27^ Although traditional capillaroscopy is limited to the nailfold, OCTA can be used to assess the effects of SSc in the retina, nailfold, skin, and other parts of the body commonly associated with SSc progression, which may better capture the onset and progression of SSc.

In this study, we first utilized OCE to evaluate the changes in murine skin *in vivo* longitudinally to assess the efficacy of an SSc-like fibrosis model. The mice were divided into two groups of six mice (i.e., control and dermal fibrosis). We observed that OCE was able to detect changes in stiffness starting at week 1, but the changes were only significant in week 4. Following this study, we employed multifunctional OCT (encompassing OCT, OCE, and OCTA) to non-invasively evaluate morphological, mechanical, and vascular changes in a murine model of dermal fibrosis akin to SSc with a single imaging system. Mice were divided into three groups (control, untreated dermal fibrosis, and treated dermal fibrosis), and we observed distinguishable differences across these groups. Our results emphasize the potential of OCT to quantitatively assess multiple aspects of fibrosis involvement in the skin entirely non-invasively for understanding SSc-induced skin changes and therapeutic outcomes.

## 2 Materials and Methods

### 2.1 Animals

A total of 24, 8-week-old, pathogen-free female C3H/HeJ mice were purchased from Jackson Laboratory (Bar Harbor, Maine, United States). The mice were divided into two batches of 12 mice each. Batch 1 was used for the longitudinal study to validate the fibrosis model. Batch 1 was further divided into two groups of six mice: (1) phosphate-buffered saline (PBS) control and (2) subcutaneous bleomycin (BLM)-treated dermal fibrosis mice. Batch 2 was used for the potential therapy study: 12 mice were divided into three groups of four mice each: (1) PBS control, (2) subcutaneous BLM-treated dermal fibrosis, and (3) BLM + imatinib-treated mice, with four mice randomly chosen for inclusion in each group. Pharmacologic grade BLM was obtained from Teva Parenteral Medicines Inc. (Irvine, California, United States) to induce dermal fibrosis.^28^ Imatinib mesylate was obtained from Sigma-Aldrich (Burlington, Massachusetts, United States) as a potential treatment for reducing dermal fibrosis.^29^ BLM was dissolved in sterile 1× PBS at a concentration of 100  μg/mL and was intradermally administered to shaved lower dorsal skin once per day (6 days a week) in a total volume of 100  μL/day for 4 weeks. Imatinib mesylate was dissolved in sterile 0.9% NaCl at a 10-mg/mL concentration. One dose of 50  mg/kg was given daily for 4 weeks. The control group received 100-μL sterile PBS daily. Children’s Tylenol was mixed with all mice drinking water (*ad libitum*) to minimize pain and reduce inflammation.

During the *in vivo* measurements, all mice were anesthetized with isoflurane (2.5% of 2  L/min), and *in vivo* OCE measurements were performed on identical locations on the lower back after hair removal. The region of interest was outlined with a permanent marker to make sure that the imaging and the injections (after imaging) were performed in the same area. The measurements included only OCE for batch 1 and OCT, OCE, and OCTA imaging for batch 2; these imaging protocols are detailed below.

After the assessment, the animals were humanely euthanized by ${\mathrm{CO}}_{2}$ inhalation, and the skin tissue was collected for histological analysis. The mice in the imatinib treatment group did not survive past the third week, so the skin was harvested in the third week. The Institutional Animal Care and Use Committee of the University of Houston approved all studies and procedures.

### 2.2 Histology Analysis

A 6-mm punch of the skin tissues was fixed in a 10% formalin solution and embedded in paraffin blocks. The 5-μm thickness paraffin sections were prepared and stained with hematoxylin and eosin (H&E) to evaluate histopathological changes and Masson’s Trichrome stain to evaluate collagen accumulation in the dermis. The dermal thickness was defined from the straight distance measurement of the dermal-epidermal junction to the dermal-subcutaneous fat junction by Image J software utilizing light microscopy images of the H&E. Histological analysis was assessed by investigators who were blinded to the sample types.

### 2.3 Phase-Stabilized Swept Source Optical Coherence Tomography System

**Fig. 1:**
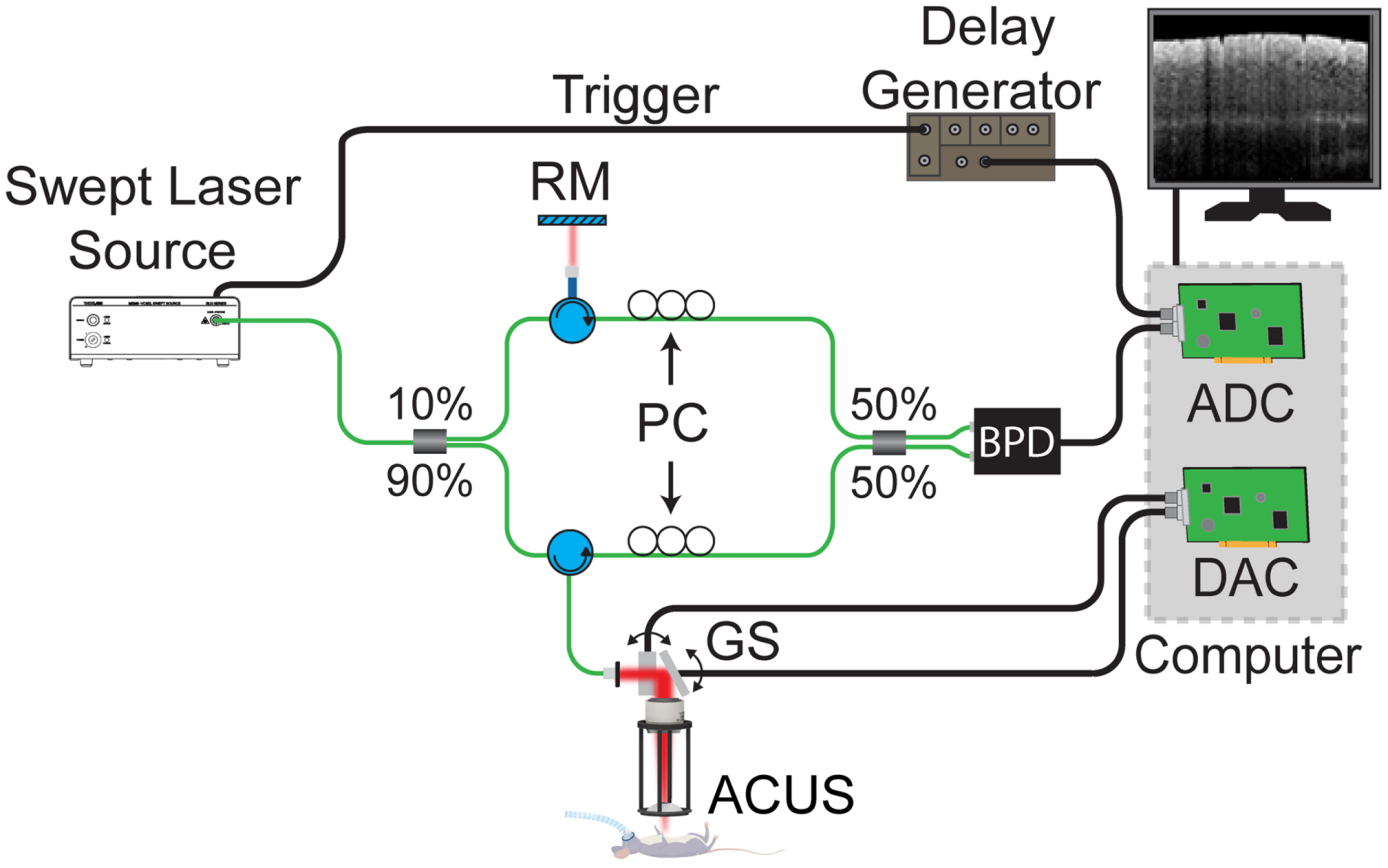
Multifunctional OCT system schematic. ADC, analog to digital converter; BPD, balanced photodetector; DAC, digital to analog converter; GS, galvanometer scanners; RM, reference mirror; ACUS, air-coupled ultrasound transducer; PC, polarization controller.

The OCT system was based on a swept source laser (SL131090, Thorlabs Inc., New Jersey, United States) with a central wavelength of 1300 nm, a bandwidth of 108 nm, and a sweep rate of 100 kHz. The OCT system was based on a Mach–Zehnder interferometer, as shown in Fig. 1. Polarization controllers in the sample and reference arms were utilized to maximize the signal-to-noise ratio (SNR) of the OCT image. The sensitivity of the OCT system was measured as 104.2 dB, and the sensitivity roll-off was 2 dB over 3.2 mm. The axial resolution of the system was measured as 8.46  μm in air and a transverse resolution of 13.9  μm with an LSM03 objective lens (Thorlabs Inc.). The total imaging depth of the system was 7.2 mm in air. The power of the OCT beam incident on the sample was 9.5 mW.

### 2.4 OCT Signal Slope

Three-dimensional OCT imaging was performed at the lower dorsal region of the mice, i.e., the location of the injection. The scan consisted of 1000 B-scans per volume and 1000 A-scans per B-scan, covering an area of 6.12 mm by 5.56 mm. Five B-scans were recorded at each B-scan position for averaging to improve the image quality and for angiographic imaging, which is detailed below. The time for each B-scan was 10 ms, and the total acquisition time was ∼1  min, including the scanner flyback time between B-scan. The OCT signal slope (OCTSS) was calculated for each of the averaged five OCT images at the center (region highly affected by medications) acquired per sample per imaging session. Intensity-based thresholding tracked the boundaries, i.e., the top surface and the noise floor of the image. The signal slope was only calculated in the dermis region. A least squares linear fit was performed for each A-line, and the final OCTSS value was obtained from a sample-wise average of the linear fits. In samples where the EDJ could not be visualized, the average dermis depth from the other samples in this study was utilized as the starting depth of the dermis. Again, the fit was performed on the OCT A-lines from this depth to the noise floor.

### 2.5 Optical Coherence Elastography

For mechanical excitation, a hemispherical air-coupled ultrasound transducer (ACUS) was used for inducing mechanical waves non-invasively in the skin.^30^ ACUS was employed to provide a noncontact method for mechanical excitation. Given that bleomycin-induced fibrosis results in increased skin fragility in mice—and considering that SSc patients may present with sensitive or ulcerated skin—a noncontact approach was chosen to avoid further tissue damage and to ensure a consistent, reproducible excitation for biomechanical assessment. The transducer resonant frequency was 1 MHz, with a focal distance of 20 mm and a lateral spot size of ∼0.3  mm. A signal generator produced a continuous 1-MHz sinusoidal signal, which was amplitude-modulated by a square 1-kHz pulse train consisting of multiple pulses. This quasi-harmonic excitation scheme combined with a bandpass filter further improved signal isolation from physiological motion, e.g., breathing and heartbeat. A robust fit was utilized to track the wave in space-time maps for measuring velocity. The large number of positions and robust linear fitting (i.e., residual-weighted linear regression) meant that this motion did not significantly influence the measured wave speed. This signal was then amplified and drove the ACUS transducer. In contrast to the previously utilized air pulse,^22^^,^^23^^,^^31^ ACUS has tight control of the excitation, enabling the generation of quasi-harmonic loading. OCE imaging was performed with the M-B-mode protocol.^32^^,^^33^ Here, 1000 successive M-mode images were acquired over 6.12 mm (x-direction) and 5.56 mm (y-direction), where the excitation was at the center of the scanned lines. Each M-mode image was 1000 A-lines (10 ms). By synchronizing the ACUS with the M-mode frame trigger, the OCT system effectively imaged the elastic wave propagation with a framerate of 100 kHz.^14^^,^^32^^,^^33^ Two separate OCE measurements were performed across the lateral-medial (x-axis) and rostral-caudal (y-axis) axes centered at the injected area on the lower dorsal region of the mice.

The wave speed was quantified from the axial particle velocity, $v_{z}$, based on the depth-dependent phase difference, Δφ(z,t), of two consecutive complex value A-lines for a given position $\left(x_{0},y_{0}\right)$^34^ using

$$v_{z}=\Delta \varphi (z,t)\frac{\lambda_{0}}{4\pi n\Delta t},$$ (1)

where n was the refractive index of the sample (n=1.42^35^^,^^36^), Δt was the temporal resolution (10  μs), Δφ(z,t)=φ(z,t+Δt)−φ(z,t), and $\lambda_{0}$ was the central wavelength of the OCT system (1300 nm). A spatiotemporal image of the wave propagation was temporally filtered using a bandpass filter at the excitation frequency (1 kHz). To get better SNR, the spatiotemporal image was generated by averaging in depth, i.e., 170  μm from the surface, and the elastic wave velocity was determined by computing the slope of the spatiotemporal images of the wave propagation.^34^

### 2.6 Vasculature Imaging

The vasculature maps were obtained using correlation mapping optical coherence angiography (cm-OCA) algorithm^37^ based on the same data acquired from the 3D OCT structural images. As five B-scans were acquired per position, vasculature imaging could be performed on this same set of data. A discrete Fourier transform–based sub-pixel registration technique^38^ was used to correct the axial and lateral shifts caused by bulk motion between B-scans recorded at the same spatial position. SNR-dependent artifacts were corrected using the temporal variance of the background noise as a function of imaging depth.^37^ The correlation of pixels between B-scans was calculated, averaged for B-scan pairs, and mapped. Angiograms with a global correlation value below a threshold of the difference between the mean and the standard deviation were disregarded. The 3D vasculature maps were obtained from the spatial distribution of the temporal correlation coefficients of the entire 3D image. A frequency rejection filter^39^ was applied to the 2D images to remove bulk motion artifacts due to respiration. The maximum intensity projection was then utilized for presentation as well as quantifications. The diameter of the lumen of the largest vessel in the imaged field of view was quantified utilizing QuPath (UK).^40^

### 2.7 Statistical Analysis

The results are expressed as inter-sample means ± standard deviation. Box and whisker plots show the interquartile range as the box, the central line in the box is the median, the whiskers are the 5^th^ and 95^th^ percentiles, and the central inscribed box is the mean. The sample-wise data points are also plotted. Significance is determined using a two-sample t-test as we have treatment groups that were independent of one another, and a p-value of <0.05 is considered significant.

## 3 Results

### 3.1 Longitudinal Study of the Fibrosis Model

**Fig. 2:**
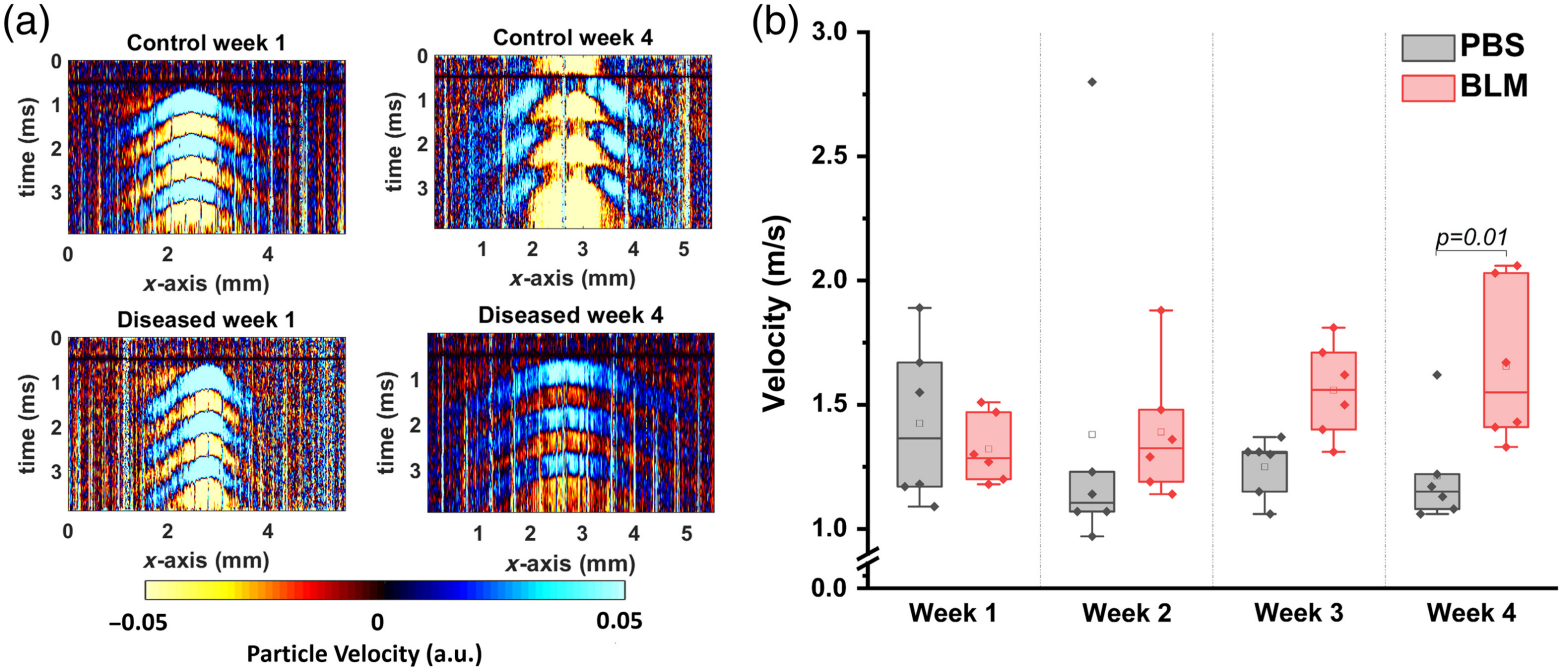
(a) Space-time maps from typical control and fibrotic (BLM) samples at weeks 1 and 4. (b) Elastic wave velocity in the different groups data from disease progression study. Mice received subcutaneous injections of bleomycin or PBS (control) for 4 weeks. Elastic wave velocity in the different groups data from disease progression study. Mice received subcutaneous injections of bleomycin or PBS (control) for 4 weeks. Elastic wave velocity was measured by OCE every week for 4 weeks. N=6 per group. Two-sample t-tests were used to test for a significant difference between the groups.

The OCE results showing the progression of skin fibrosis are plotted in Fig. 2. The OCE data were taken on rostral-caudal and lateral-medial axes, and the resulting wave speeds were averaged. The PBS-injected control skin was significantly softer (p=0.01, mean wave speed of 1.2±0.2  m/s) than the BLM-injected (mean wave speed=1.6±0.3  m/s) fibrotic skin as quantified by a two-sample t-test on week 4 (day 28), as plotted in Fig. 2(a). This difference in skin stiffness was not fully manifested until week 4, which was the selected time point for the multifunctional study focused on assessing the effects of the potential anti-fibrotic treatment.

### 3.2 Anti-Fibrosis Response Study

**Fig. 3:**
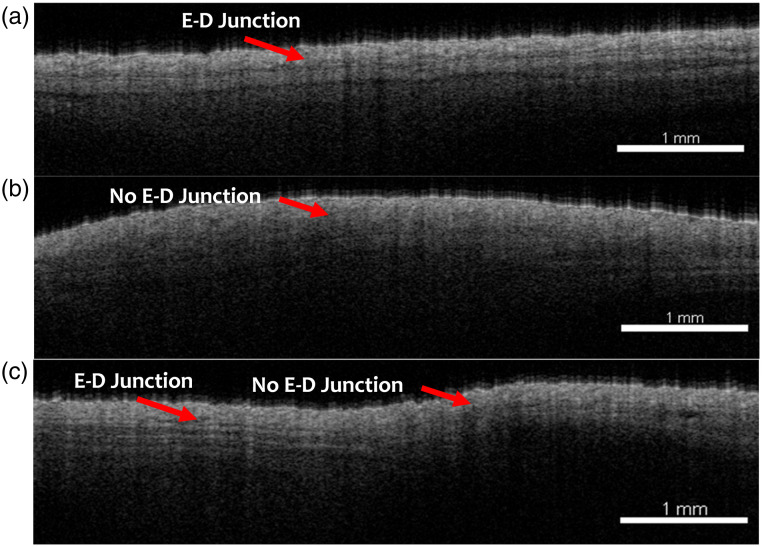
OCT structural images of mouse skin from (a) PBS-injected, (b) BLM-injected, and (c) BLM + imatinib-injected skins of the 4-week treatment. N=4 per group, representative images.

One hallmark of SSc is the disappearance of the EDJ, which is evident in the representative OCT structural images from each group of mice. The EDJ is visible in the control mice that were injected with only PBS [Fig. 3(a)]. In the BLM-injected fibrosis group, there was no clear demarcation between the epidermis and dermis [Fig. 3(b)]. However, in the BLM and imatinib treatment group, the EDJ was apparent in some parts of the skin but was absent in other locations [Fig. 3(c)]. The superior resolution and contrast of OCT make it well-suited for imaging the EDJ.

**Fig. 4:**
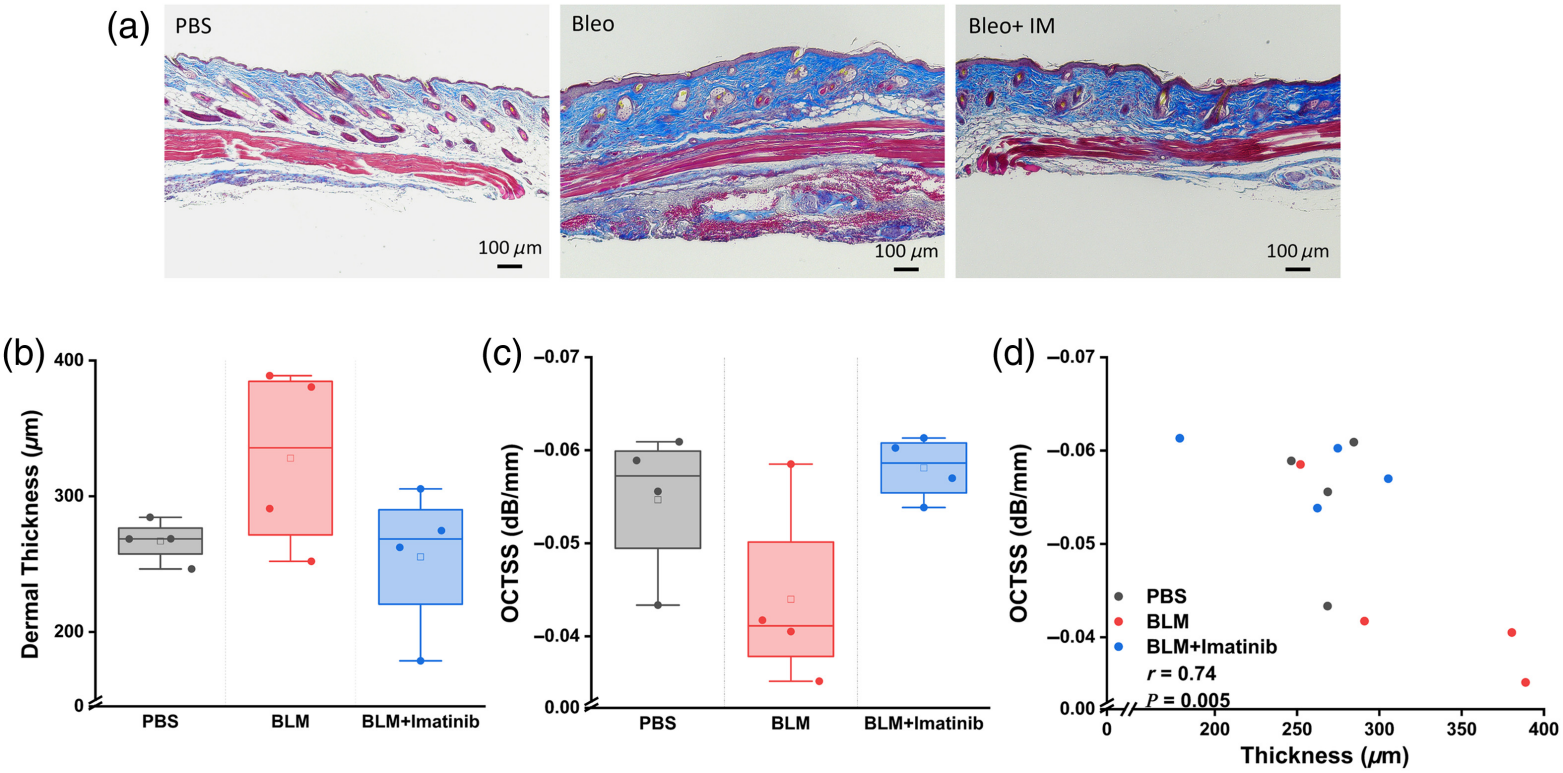
(a) Masson’s trichrome stain images from all three groups. (b) Dermal thickness measurement in histology. (c) OCTSS. (d) Correlation analysis between histological dermal thickness and OCTSS.

Dermal thickness was measured in histology images, and typical results are shown in Fig. 4(a). The BLM-injected mouse skin (mean dermal thickness=328.1±67.3  μm) showed a trend increase in skin thickness, which was not significant compared with the controls (mean dermal thickness=267±15.6  μm). The dermal thickness of the imatinib group (mean dermal thickness=255.3±54.1  μm) showed impaired dermal thickness compared with the BLM-injected group and was similar to the control group, as plotted in Fig. 4(b). In addition to visualizing the EDJ, the OCT images were also used to calculate the OCTSS,^41^ which reflects the scattering properties of tissue. The OCTSS results in Fig. 4(c) showed a decrease in OCTSS for the BLM-injected (mean OCTSS=−0.044±0.01  dB/mm) mice but no statistically significant difference to the control group (mean OCTSS=−0.057±0.008  dB/mm). The OCTSS results for the treatment group (mean OCTSS=−0.058±0.003  dB/mm) showed a similar trend to the control group. The OCTSS results in Fig. 4(c), similar to dermal thickness results in histology, showed no significant difference between groups. Pearson’s correlation between the histological dermal thickness and OCTSS was r=0.74 and statistically significant (p=0.005).

**Fig. 5:**
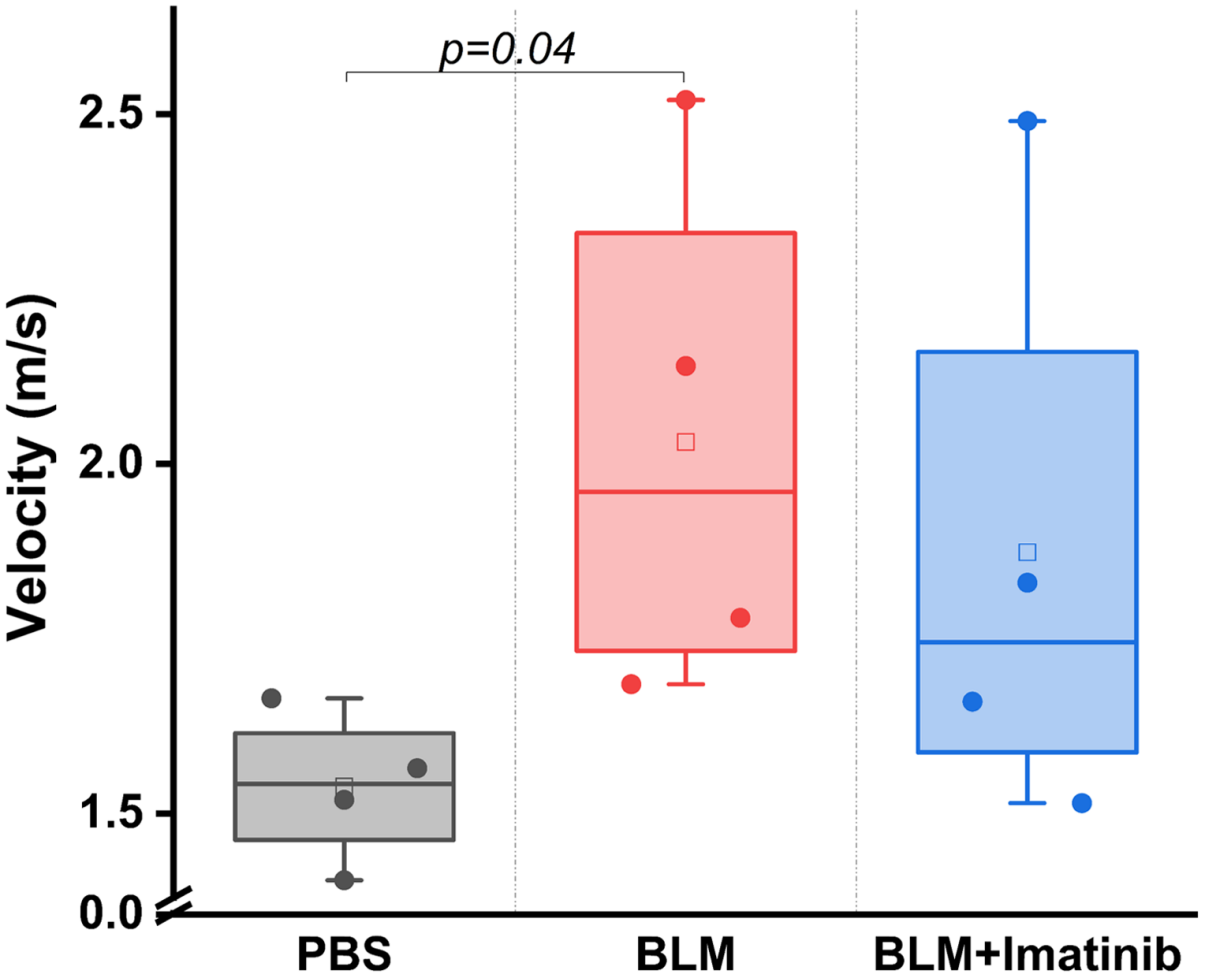
Velocity of the elastic wave in the different groups.

The OCE results from the PBS controls, bleomycin-treated fibrotic group, and bleomycin and imatinib-treated groups are plotted in Fig. 5. The OCE data were taken on rostral-caudal and lateral-medial axes, and the resulting wave speeds were averaged. The PBS-injected control skin was shown to significantly lower speed velocity (skin softener) (p=0.04, mean OCE wave speed=1.5±0.1  m/s) compared with the BLM-injected fibrotic skin (mean OCE wave speed=1.9±0.3  m/s) as quantified by a two-sample t-test. The imatinib treatment showed a trend toward fibrotic skin softening compared with the BLM-injected mice skin, but it was not significant (p=0.18, mean OCE wave speed=1.7±0.4  m/s).

**Fig. 6:**
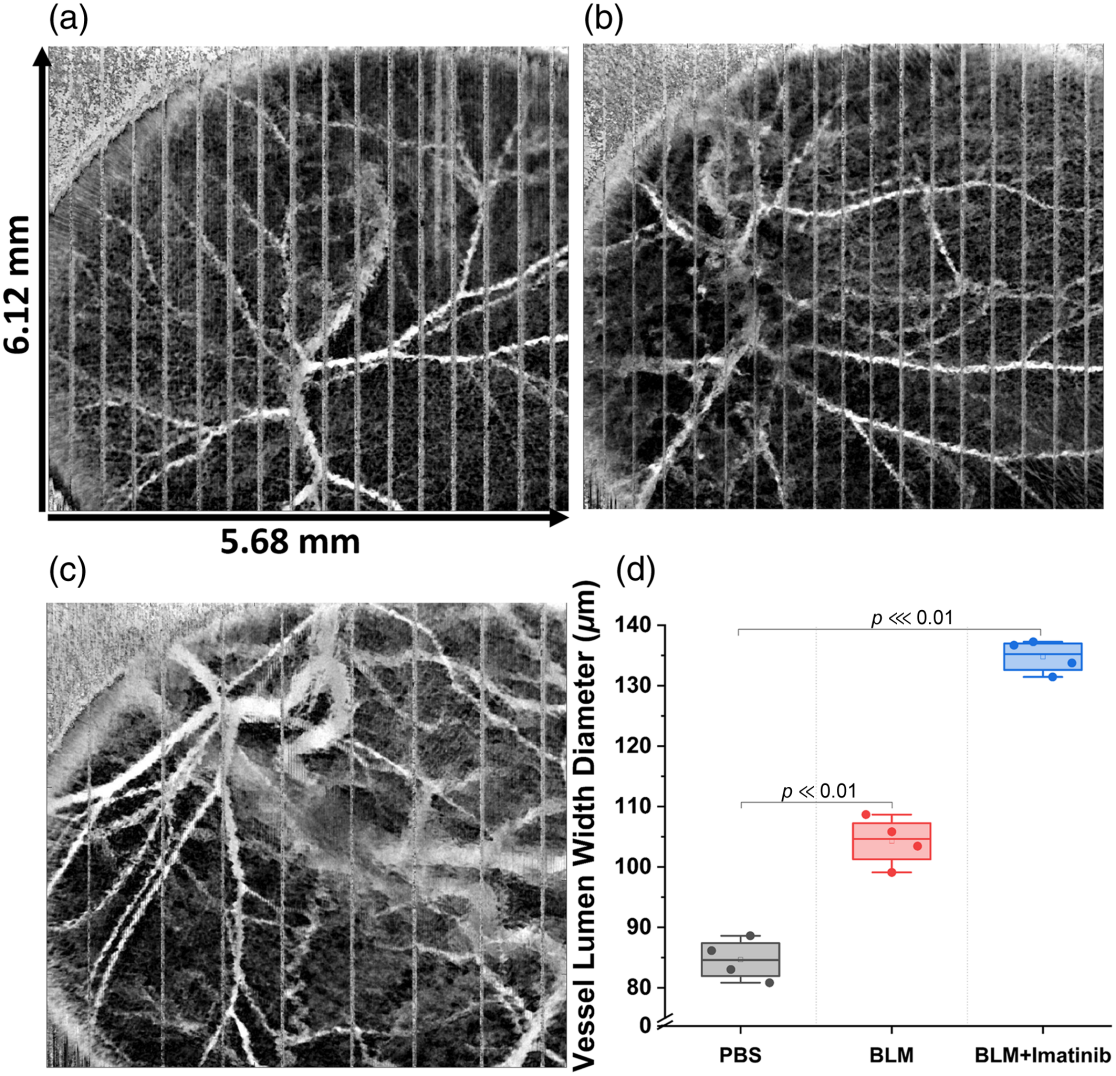
Maximum intensity projections of the 3D cm-OCA data from a typical sample that was (a) PBS-injected, (b) BLM-injected, (c) BLM + imatinib-injected. (d) Lumen diameter of the largest vessel in the field of view from all three groups.

OCTA results are shown in Fig. 6; the stripes in the images are due to breathing motion that could not be corrected due to the large amplitude of the motion and nonlinear movement. During vessel lumen diameter quantification, these stripes were ignored. The results show that the lumen diameter was significantly larger for the diseased (mean lumen width=104.6±4  μm) and treatment groups (p⋘0.01, mean lumen width=134.7±2.7  μm) compared with the control group (p≪0.01, mean lumen width=84.6±3.4  μm).

## 4 Discussion

This study demonstrates the capability of multifunctional OCT to quantify various parameters of skin fibrotic conditions in a murine model *in vivo*. This model employs subcutaneous injections of BLM, which elicits local inflammation and fibrosis in the skin, mimicking human SSc skin manifestation.^42^^,^^43^ Another method for inducing fibrosis involves the use of hypochlorous acid, but this model is chosen for acute fibrosis. As we wanted to assess the longitudinal changes in the skin due to fibrosis mimicking SSc involvement in the skin, which occurs over a much longer period, the bleomycin model was used. This study also utilized multifunctional OCT to quantitatively assess the anti-fibrotic effects of imatinib in a murine skin fibrosis model.

### 4.1 Longitudinal Study of the Fibrosis Model

The longitudinal study demonstrated that OCT effectively detected subtle variations in skin stiffness between the control group (PBS-injected) and the fibrotic skin group (BLM-injected). This finding validates our model of SSc-like fibrosis in the skin. Other researchers have reported comparable results, indicating that bleomycin typically induces fibrosis in mice within a similar 4-week timeframe.^44^

### 4.2 Anti-Fibrosis Response Study

#### 4.2.1 Morphological changes EDJ and OCTSS

After establishing the mechanical phenotype of the SSc-like fibrosis *in vivo*, multifunctional OCT captured several different features of the skin to evaluate the SSc-like conditions in the murine model. To detect morphological changes, 3D OCT structural images of the affected area were acquired to visualize the presence or disappearance of EDJ, which is one of the biomarkers for SSc because the increased fibrosis in the skin can cause the EDJ to disappear in SSc.^45^ The absence of the EDJ in the skin of patients with SSc represents a notable histopathological characteristic with substantial implications. This phenomenon may result in compromised skin functionality,^46^ modifications in vasculature,^47^ and altered cellular interactions.^47^^,^^48^ Furthermore, it may serve as a biomarker for disease progression and is correlated with an increase in collagen accumulation.^46^^,^^49^ OCT is well-suited to image this feature due to its high spatial resolution, depth penetration, and superior contrast compared with other imaging methods. The results followed our initial hypotheses that the EDJ would disappear in the fibrotic mouse skin in the BLM-treated group, which corroborates with previous research.^45^ Interestingly, the EDJ was heterogeneous in the treatment group, indicating that the fibrosis was not fully reduced at the time of the final imaging session 3 weeks after the treatment began. Imatinib reduces fibrosis by multiple mechanisms, such as inhibition of fibroblast activation, reduction of extracellular matrix production, and decrease in transforming growth factor-beta (TGFβ) activity.^50^^–^^52^

The next feature quantified in the present multifunctional OCT technique was the scattering properties of the tissue. OCT relies on the backscattering of light within tissue to obtain its depth-resolved information,^11^ so it is a powerful tool for assessing tissue optical properties. In SSc, excess collagen accumulation in the skin can alter the scattering properties of the tissues. One added benefit of multifunctional OCT imaging is that many skin properties can be imaged in one session with one instrument. Thus, additional imaging was not needed to obtain the optical scattering properties of the tissue. Although our results in this mouse model showed an increase in light scattering, these changes were not statistically significant, even though previous results have shown significant differences in the scattering properties of the skin in a mouse model^31^ and SSc patients.^22^^,^^45^ Similarly, close results were obtained for histological dermal thickness. The distribution of results suggests that the lack of statistical significance for both OCTSS and dermal thickness results is likely due to a low sample size. Nevertheless, there was a strong (r=0.74) and significant (p=0.005) negative correlation of OCTSS with histological skin thickness measurements, indicating that there was a notable relationship between fibrosis and OCTSS. Our future work is focused on implementing more robust scattering quantification protocols that can provide quantitative metrics of tissue scattering properties^53^ and utilizing higher resolution OCT systems for future studies. Conversely, the mRSS offers only a ranking of the patient’s skin elasticity based on palpation. By contrast, OCE addresses all the limitations of mRSS by delivering more quantitative results rather than mere rankings. Furthermore, biomarkers such as the EDJ and additional quantitative measurements such as the OCTSS, as well as changes in vasculature, cannot be assessed using mRSS.

#### 4.2.2 Mechanical changes

Currently, mRSS, which relies on manual palpation, is the reference standard to assess skin stiffness and thickness in SSc. However, the mRSS uses an ordinal scale of 0 to 3 and can have poor inter-observer reliability.^54^ To overcome these limitations, we characterized the mechanical properties of murine skin *in vivo* longitudinally with OCE to characterize the dynamic manifestation of the mechanical phenotype and as a part of the multifunctional OCT imaging to assess the effects of a potential anti-fibrotic treatment. OCE can quantitatively measure skin involvement in SSc with greater repeatability, accuracy, and precision than the mRSS, as demonstrated in a small cohort of normal subjects and SSc-afflicted patients.^23^ The OCE results show that the wave speed for the SSc-afflicted skin was greater than in the controls, as plotted in Figs. 2 and 5, indicating an increase in the stiffness of fibrotic tissue compared with the control skin. This correlates well with the previously conducted studies using OCE,^22^^,^^55^ ultrasound elastography,^56^ and the Vesmeter.^17^ This increase in stiffness is the result of increased collagen accumulation in the skin. The results also show that the diseased group (BLM only) and the treatment group (BLM + imatinib) had a large variance in the wave speed values, which may depend on variations of the skin fibrosis progression and collagen deposition in different mice. This is a well-known effect in SSc.^57^ Moreover, the stiffening of the skin only manifested as a significant difference at a 4-week time point after BLM administration, which corroborates with previous studies on the skin.^42^ In addition, the wave speeds in the treated fibrosis group were in between that of the control and diseased samples, suggesting that imatinib can prevent skin stiffening associated with fibrosis. However, the relatively small sample size limits a strong conclusion. Although our OCE results showed a significant difference in the wave speed in the control and fibrotic samples, the wave speed can also be affected by parameters other than intrinsic mechanical properties. For example, the skin thickness and boundary conditions during the OCE measurement can impact the measured wave speed.^58^ Our future work is focused on utilizing more robust mechanical models that incorporate the layered geometry of the skin and higher excitation frequencies to isolate the mechanical properties of the skin and reduce the influence of boundary conditions.^59^^,^^60^

#### 4.2.3 Vascular changes

The final parameter captured during multifunctional OCT imaging was the vessel lumen diameter via OCTA. Here, repeated images were acquired in the same location, and any minute motion detected between consecutive scans was assumed to be due to blood flow. Hence, the correlation of a given pixel over the repeated scans was calculated, and the inverse of the correlation was displayed, which appears as the contrast of motion from blood against a static background of other tissues.^37^ OCTA has become a powerful tool for detecting disease in ophthalmology,^61^ and our results show that OCTA can also detect changes in dermal vasculature due to localized fibrosis in mice *in vivo*. Here, the OCTA results show significant vessel dilation in the fibrosis and treatment groups compared with the control group. The results for the fibrotic group correlate with a previous SSc study utilizing nailfold capillaroscopy in SSc patients.^62^ Our results show that the treatment group had even greater dilation in their dermal vasculature. Many studies have been conducted that show vasculature changes in humans with SSc.^27^^,^^63^ However, the results cannot be correlated with these studies due to the different effects of fibrosis caused by mice skin using bleomycin. Nevertheless, OCTA shows strong potential for monitoring these changes in the clinic.

#### 4.2.4 Limitations of the current study

Imatinib is a tyrosine kinase inhibitor used to treat solid tumors by inhibiting cell growth and division.^64^ Imatinib reduces fibrosis by inhibiting platelet-derived growth factor and TGF-β pathways, which decreases fibroblast activation and collagen production.^65^ It prevents fibroblasts from becoming myofibroblasts, which are critical players in fibrosis, limits their proliferation,^66^ and reduces stiffness and thickness of the dermis in the skin. Imatinib dilates blood vessels by inhibiting PDGF receptors in vascular smooth muscle cells, leading to muscle relaxation. It also reduces endothelin-1, a vasoconstrictor, and may enhance nitric oxide (NO) signaling, promoting vasodilation.^67^ These effects might be reflected in our results for vessel lumen width.

The BLM-induced SSc-like fibrosis model shows significant similarities with SSc in patients,^28^ supporting the clinical value of the presented results. On the other hand, the treatment response results (imatinib group) were inconclusive as the mice grew very weak in the third week, 1 week before the study completion period of 4 weeks. Hence, the data shown for the treatment group are from week 3 (1 week before the completion period). Thus, the fibrosis in this treatment group may have advanced further in the additional week compared with the control and fibrosis groups, as shown in Fig. 5. As previously stated, imatinib is frequently utilized in the treatment of cancer and may induce considerable fatigue as it restricts cellular division and proliferation. This phenomenon was evident in the notable weight reduction and mortality observed within the imatinib study cohort by the third week, where the treatment was poorly tolerated by the participants. The loss of the treatment group was a major limitation for the overall analysis of study results. Thus, our future work is focused on assessing alternative therapies for SSc that can be better tolerated and are perhaps targeted anti-fibrotic agents, such as TGFβ inhibitors.^68^ In addition, this part of the study was limited by a small number of subjects (n=4 per group for response study). Future work will expand this study to include additional subjects per response group for a more meaningful evaluation of therapy response.

### 4.3 Multifunctional OCT

Overall, our results show that multifunctional OCT can detect and quantify morphological, mechanical, and vasculature changes in the fibrotic skin of the murine model that simulates SSc. The disappearance of the EDJ, stiffening of the skin, and vessel dilation may be due to the increased fibroblast activity and associated collagen deposition in the skin. Moreover, multifunctional OCT was used to assess the efficacy of a potential anti-fibrotic treatment, and the results showed that the EDJ was still present in some samples, and the skin stiffness was reduced compared with the diseased samples. However, the treatment caused dramatic vasodilation compared with the diseased group, which would not have been captured by structural or elasticity imaging alone. An additional benefit of multifunctional imaging is the potential for robust artificial intelligence (AI) to classify tissue health, and our previous work has shown that integrating multiple parameters obtained from OCT and OCE imaging improved the accuracy of tissue health classification by ∼20% compared with OCE or OCE imaging alone.^69^ Machine learning models such as ensemble bagged trees, ensemble RUSBoosted trees, and linear support vector machines can use these multiple parameters and increase the accuracy, sensitivity, and specificity of the assessment.^70^^,^^71^ This is a significant avenue of our future work to integrate OCT, OCE, and OCTA parameters into AI models for more robust disease classification and staging. Although a similar approach of using OCT and its extensions has been used to study tumors,^70^ all the extensions have never been used to study fibrosis. Multifunctional OCT has the potential to enhance the assessment of SSc due to the absence of quantitative methodologies in current practices. In addition, multiple parameters can provide more details for assessing complex diseases like SSc.

## 5 Conclusion

OCT is a powerful, non-invasive technique for imaging tissues in 3D with a micrometer-scale resolution, and its utility can be significantly expanded with functional extensions, such as mechanical and angiographic imaging, as demonstrated in this work. In this study, we showed the capability of multifunctional OCT to detect fibrosis *in vivo* in a murine model of SSc. Our results showed that multifunctional OCT was able to detect morphological, mechanical, and vasculature changes in the murine skin caused by bleomycin and can detect differences in these tissue properties due to therapeutic intervention. We measured (1) the disappearance of the EDJ and changes in scattering properties of the skin, (2) small changes in skin stiffness that distinguish different groups, and (3) vessel lumen width dilation in the murine skin caused by bleomycin. Thus, multifunctional OCT could be a powerful tool for quantitative measurements of various skin properties in SSc, overcoming the significant limitations of current clinical standards for SSc assessment. Future work will expand to clinical studies, including the detection and staging of SSc, and will further be used to evaluate the efficacy of additional therapies.

## Data Availability

The data and computer code supporting this study’s findings are available from the corresponding author upon reasonable request.
