# Assessment of skin fibrosis in a murine model of systemic sclerosis with multifunctional optical coherence tomography (Erratum)

**DOI:** 10.1117/1.JBO.31.1.019801

**Published:** 2026-01-23

**Authors:** Harshdeep Singh Chawla, Yanping Chen, Minghua Wu, Pavel Nikitin, Jessica Gutierrez, Chandra Mohan, Manmohan Singh, Salavat R. Aglyamov, Shervin Assassi, Kirill V. Larin

**Affiliations:** a University of Houston, Biomedical Engineering, Houston, Texas, United States; b University of Texas Health Science Center at Houston (UTHealth Houston), Division of Rheumatology, Department of Medicine, Houston, Texas, United States; c University of Houston, Mechanical and Aerospace Engineering, Houston, Texas, United States; d Baylor College of Medicine, Integrative Physiology, Houston, Texas, United States

## Abstract

The erratum corrects a misstatement in the Introduction section of the published article.

This article [*J. Biomed. Opt.* **30**(3), 036007 (2025), doi: 10.1117/1.JBO.30.3.036007] was originally published on 27 March 2025 with a misstatement in the Introduction section.

The original sentences on page 2 stated, “A device called the Vesmeter can assess skin thickness and stiffness based on suctioning the skin and has been utilized for characterizing SSc involvement in the skin. However, this device requires specific pressure and point-wise measurements, which introduces significant variability.”

The authors acknowledge that the Vesmeter operates on an indentation-based principle rather than suction. The sentences have been revised accordingly as follows: “A device called the Vesmeter can assess the elasticity and viscosity of skin based on an indentation-based principle and has been utilized for characterizing SSc involvement in the skin. However, this device requires specific pressure and point-wise measurements, which introduces approximately 20% inter-observer variability.”

The misstatement and the correction do not affect the scope or the conclusions of the published article. However, the article was corrected and republished 10 January 2026 to ensure technical accuracy.

